# Pheochromocytoma associated with neurofibromatosis type 1: concepts and current trends

**DOI:** 10.1186/1477-7819-8-14

**Published:** 2010-03-10

**Authors:** George N Zografos, George K Vasiliadis, Flora Zagouri, Chrysanthi Aggeli, Dimitris Korkolis, Sophia Vogiaki, Matina K Pagoni, Gregory Kaltsas, George Piaditis

**Affiliations:** 1 Third Department of Surgery, G. Gennimatas Hospital, Athens, Greece; 2 Department of Clinical Therapeutics, Alexandra Hospital, Athens, Greece; 3 Department of Internal Medicine, G. Gennimatas Hospital, Athens, Greece; 4 Department of Endocrinology, G. Gennimatas Hospital, Athens, Greece

## Abstract

**Background:**

Neurofibromatosis Type 1(NF-1) has autosomal dominant inheritance with complete penetrance, variable expression and a high rate of new mutation. Pheochromocytoma occurs in 0.1%-5.7% of patients with NF-1.

**Case presentation:**

We present the case of a 37-year-old patient with laparoscopically resected pheochromocytoma. He was investigated for hypertension, flushing and ectopic heart beat. Abdominal CT and MRI revealed a mass measuring 8 × 4 cm in the right adrenal gland. The diagnosis of pheochromocytoma was confirmed by elevated 24-hour urine levels of VMA, metanephrines and catecholamines as well as positive MIBG scan. The patient presented with classic clinical features of NF-1, which was confirmed by pathologic evaluation of an excised skin nodule. The patient underwent laparoscopic right adrenalectomy through a transabdominal approach and was discharged on the second postoperative day, being normotensive. The patient is normotensive without antihypertensive therapy 11 years after the procedure.

**Conclusion:**

Nowadays in the era of laparoscopy, patients with pheochromocytoma reach the operating theatre easier than in the past. Despite, the feasibility and oncological efficacy of the laparoscopic approach to the adrenals, continued long term follow-up is needed to establish the minimally invasive technique as the preferred approach. Furthermore, these patients should be further investigated for other neoplasias and stigmata of other neurocutaneous syndromes, taking into account the association of the familial pheochromo-cytoma with other familial basis inherited diseases.

## Background

Pheochromocytomas are tumors of the adrenal medulla and extra-adrenal chromaffin tissue that secrete catecholamines, resulting in hypertension, whether sustained or paroxysmal, and other symptoms of increased production of catecholamines [1-3]. They may be classified as sporadic or familial. Most of the pheochromocytomas are sporadic [4]. Familial predisposition is seen mainly in patients with multiple endocrine neoplasia type 2, neurofibromatosis Type 1 (NF-1), von Hippel-Lindau disease and familial carotid body tumors [5].

NF-1 affects approximately 1 in 3500 individuals worldwide and it has autosomal dominant inheritance with complete penetrance [6]. Pheochromocytoma occurs in 0.1%-5.7% of patients with NF-1 [7]. The only treatment of pheochromocytoma is surgical removal [1]. The traditional open approach (either transabdominal, or retroperitoneal) has been replaced by the laparoscopic transabdominal and the endoscopic retroperitoneal procedure.

Despite the feasibility and oncological efficacy of the laparoscopic approach to the adrenals, continued long-term follow-up is needed to establish the minimally invasive technique as the preferred approach [8,9]. Herein, we present a case of a patient with NF-1 and pheochromocytoma, who was successfully treated with laparoscopic right adrenalectomy 11 years ago.

## Case presentation

A 37-year-old patient in good general condition (height: 175 cm, weight: 70 kg), was admitted to our hospital for surgical treatment of a diagnosed pheochromocytoma. The patient reported that during last year the family physician had repeatedly diagnosed hypertensive episodes, accompanied by intermittent attacks of severe flushing, perspiration and ectopic heart beat. During the hospital stay blood pressure was closely monitored, fluctuating between 125/80 and 190/110 mmHg. The ECG revealed sinus arrhythmia.

Clinical examination revealed liver enlargement, *café-au-lait *spots and skin nodules. NF-1 was confirmed by pathologic evaluation of an excised skin nodule which revealed a neurofibroma. The medical history revealed pulmonary tuberculosis during childhood, which was treated with six month multiple drug therapy. Computerized tomography of the abdomen showed a round tumor, 8 × 4 cm in diameter, which could be clearly delineated from the right kidney. The I131 MIBG scan revealed the presence of chromafinic tissue only in the right adrenal.

Laboratory tests revealed elevated catecholamine, metanephrine and vanillylmandelic acid (VMA) levels in the 24-h urine collection (Table 1). The plasma level of adrenaline and cortisone were within normal limits. Thyroid and parathyroid ultrasonography was normal. Serum calcitonin levels were 8.5 pg|ml (normal range: 8-10 pg/ml), whereas serum parathormone levels were 18.3 pg|ml (range: 8-76 pg/ml). Pituitary CT scan was normal.

**Table 1:** 24 hour urinary metabolites level

	Sample Value	Normal Value
Catecholamines	808 μg	14 - 108

Metanephrines	3.5 mg	< 1

VMA	36 mg	1.8 - 6.7

The patient preparation for the operative procedure with alpha-blocking agents started ten days before the procedure and with beta-blockers three days before it. A pacemaker was inserted to control the heart rate during the procedure. Laparoscopic adrenalectomy was performed with transabdominal approach. The patient was placed in a left lateral position. The intraoperative monitoring included continuous ECG control, invasive blood pressure recording, pulse oximetry and capnography. During the mobilization of the tumor and prior to the adrenal vein ligation, arterial blood pressure rose to 250/130 mmHg, whereas blood gases remained normal. The hypertension was treated with continuous intravenous infusion of nitroglycerine (0.1-0.5 mg/min) and hydralazine 5 mg increments. The patient was extubated immediately after the operation and transferred to a surgical ward monitored with noninvasive blood pressure monitor. He was discharged a day after.

The pathological evaluation of the tumor confirmed the diagnosis of pheochromocytoma. Urine catecholamines, metanephrines and VMA returned to normal on the 7th postoperative day. The patient is normotensive without antihypertensive therapy 11 years after the procedure.

## Discussion

Pheochromocytoma is found in 0.1% among those tested because of hypertension [1] and in 4% of patients with adrenal incidentaloma [2]. The incidence in the general population is estimated to be 1 per 100,000 persons per year or less [3] and approximately 10% of patients have bilateral pheochromocytomas [4]. Moreover, approximately 10% of pheochromocytomas are malignant at diagnosis; however this is not determined at the time of diagnosis [4]. Many malignant pheochromocytomas are found to be malignant only by the fact that they recur after a complete gross resection [4]. Most of them are sporadic. Approximately 10-15% of the cases have been thought to be due to hereditary causes [4]. Familial predisposition is seen mainly in patients with Multiple Endocrine Neoplasia (MEN) type 2, NF-1, von Hippel-Lindau disease and familial carotid body tumors (Table 2) [5].

**Table 2:** Hereditary Forms of Pheochromocytoma.

Syndrome	Frequency of Pheo (%)	Gene	Chromosome location
MEN type II	30-50	RET oncogene	10q11

VHL disease	15-20	VHL tumor suppressor gene	3p25

NF type 1	1-5	Neurofibromatosis type 1	17q11

Familial carotid body tumors		Paraganglioma	11q21-23

Unilateral or bilateral pheochromocytomas are found in 50% of patients with MEN 2 syndrome [10]. Pheochromocytomas associated with MEN 2A are diagnosed concurrently with MTC in 35-73% of the cases and as the first manifestation of MEN 2A in 9-27% of them [4]. MEN 2A patients are statistically significantly younger at age of pheochromocytoma diagnosis than patients with sporadic pheochromocytoma (mean ages of 38 and 47 years, respectively) [11-14].

NF-1 was described by Smith in 1849 and von Recklinghausen in 1882 [15]. It affects approximately 1 in 3500 individuals worldwide and it has autosomal dominant inheritance with complete penetrance, variable expression and a high rate of new mutation, approximately 50%, which is the highest rate of new mutation of any known single-gene disorder [16,6]. The NF-1 gene is a tumor suppressor gene mapping to chromosome 17q11.2. Because of the large gene size (11 kb of coding sequence extending over 300 kb of genomic DNA), mutation analysis has been difficult (in only about 15% of patients mutations are identified) [17]. The diagnosis of NF-1 is based on criteria developed by National Institutes of Health Consensus Conference in 1987 [18-20]. Pheochromocytoma occurs in 0.1%-5.7% of patients with NF-1, and in 20%-50% of NF-1 patients with hypertension, compared to 0.1% of all hypertensive individuals [7]. The mean age at diagnosis of pheochrocytoma in patients with NF-1 is 42 years [7]. Persons with NF-1 are at increased risk for malignant conditions, especially malignant peripheral nerve sheath tumor (MPNST), leukemia and rhabdomyosarcoma [17].

Von Hippel-Lindau (VHL) disease, an autosomal dominant syndrome [4], is clinically subdivided into two types: those without Pheo (VHL type 1) and those with Pheo (VHL type 2). Type 2 VHL disease, where pheochromocytoma develops, accounts for 10% of VHL disease cases [21,22]. The largest series of VHL patients with pheochromocytomas was described by Walther *et al *[23]. The mean age at diagnosis was 29.9 year, which was statistically significantly younger than the mean age at diagnosis in a control group of patients with sporadic pheochromocytoma (39.7 years)[24]. However, there may be bias due to routine screening in the VHL group [4].

The treatment of functioning adrenal tumors is surgical removal of the affected gland first described by Sargent in 1914 [25]. Since 1992 [26] laparoscopic adrenalectomy (LA) has gained field in the surgery of the adrenals and nowadays it is the procedure of choice [1,27,28]. Modern indications for LA have been expanded to large tumors, bilateral pathology and metastatic malignancies [28,29]. The feasibility and oncological efficacy of the laparoscopic approach to the adrenals have been reported in cases of malignancy; however, continued long-term follow-up is needed to establish the minimally invasive technique as the preferred approach [8,9]. The only absolute contraindication to LA currently remains large neoplastic lesions with involvement of the surrounding anatomical structures, a condition that should be treated using an open approach [27]. In this case, the tumor had intact contour, was homogeneous and based on CT scan was considered benign.

Early ligation of the central adrenal vein to facilitate pharmacologic control in pheochromocytoma has been emphasized in reports and textbooks. However, our experience and that of others indicate that early ligation of the adrenal vein is not always feasible because of the posterior entrance to the cava on the right and the medial entrance to the renal vein on the left. Our patient developed intraoperative hypertension, successfully treated. An extensive search of the literature showed that hemodynamic instability was routinely successfully treated [27,30]. From a technical standpoint, large adrenal masses are difficult to dissect laparoscopically. In our series the largest tumor laparoscopically resected was a 14 cm myelolipoma. Laparoscopic approach to large adrenal tumors, less than 15 cm in size, is feasible but necessitates experience in laparoscopic and adrenal surgery. Conversion can always be an option, and it is not considered to be a complication.

## Conclusions

In the era of laparoscopy, approach to patients with pheochromocytoma is easier than in the past. These patients should be further investigated for other neoplasias (such as thyroid carcinoma, parathyroid hyperplasia, central and peripheral nervous tumors) and stigmata of other neurocutaneous syndromes (v. Hippel Lindau disease), taking into account the association of the familial pheochromo-cytoma with other familial basis inherited diseases.

## Consent

Written informed consent was taken from the patient for publication of this case report. A copy of consent is available with editorial office.

## Competing interests

The authors declare that they have no competing interests.

## Authors' contributions

GNZ: conception and designed. GKV: manuscript preparation. CAA: data collection and manuscript preparation. MKD: data collection and manuscript preparation. GIP: data collection and manuscript preparation. DKG: data collection and manuscript preparation. MKP: data collection and manuscript preparation and review. All authors read and approved the final version.
